# Treatment Outcome of Different Chemotherapy in Patients With Relapsed or Metastatic Malignant Urachal Tumor

**DOI:** 10.3389/fonc.2021.739134

**Published:** 2021-09-15

**Authors:** Meiting Chen, Cong Xue, Ri-qing Huang, Meng-qian Ni, Lu Li, Hai-feng Li, Wei Yang, An-qi Hu, Zhou-san Zheng, Xin An, Yanxia Shi

**Affiliations:** ^1^Department of Medical Oncology, Sun Yat-sen University Cancer Center, State Key Laboratory of Oncology in South China, Collaborative Innovation Center for Cancer Medicine, Guangzhou, China; ^2^Department of Oncology, The First Affiliated Hospital of Sun Yat-sen University, Guangzhou, China

**Keywords:** urachal carcinoma, metastatic, chemotherapy, efficacy, survival

## Abstract

**Background:**

Malignant urachal tumor is a rare subtype of genitourinary cancer. Our aim was to explore the optimal chemotherapy regimens for relapsed or metastatic urachal carcinoma.

**Materials and Methods:**

We retrospectively enrolled 24 adult patients with relapsed or metastatic urachal carcinoma from January 2014 to September 2020 at Sun Yat-sen University Cancer Center. We summarized the chemotherapy regimens and classified them as fluorouracil based, platinum based, and paclitaxel based. Nine patients received XELOX (capecitabine and oxaliplatin) regimens, seven patients received TX (paclitaxel and capecitabine) regimens, and eight of them received chemotherapy including GP (gemcitabine and cisplatin), TP (paclitaxel and cisplatin), TN (paclitaxel and nedaplatin), and tislelizumab.

**Results:**

The disease control rate was 75%. Among all patients, one patient treated with XELOX achieved partial remission (PR), while 17 patients showed stable disease. The median progression-free survival (PFS) and overall survival (OS) in all treated patients was 7.43 and 29.7 months, respectively. The patients receiving first-line platinum-based chemotherapy presented better PFS than those without platinum (median PFS 8.23 *vs*. 3.80 months, *p* = 0.032), but not significant for OS between two groups. There is no significant difference in PFS and OS for fluorouracil-based and paclitaxel-based groups as first-line regimen. Next-generation gene sequencing revealed TP53 mutation and low tumor mutational burden in five out of seven cases.

**Conclusion:**

The platinum-based chemotherapy regimen is effective for relapsed or metastatic urachal carcinoma.

## Introduction

Malignant urachal tumor (MUT) is a rare genitourinary tumor derived from the urachus at the dome of the bladder, accounting for 0.1%–0.7% of all malignant bladder cancers (1). Patients with MUTs are usually diagnosed at an advanced stage with extravesical extension and lymph node metastasis, and the prognosis is generally poor (2). Literatures about MUT are mainly based on some of case reports and few retrospective studies (3–6). MUT mostly affects male patients at 50 to 60 years (3, 7). The common clinical manifestation is hematuria (8, 9). Abdominal pain and dysuria are less commonly seen. The diagnosis for MUT is difficult due to the rarity of tumor and similarity to adenocarcinoma of other origins (4, 10, 11). Several retrospective studies reported the clinicopathological features of MUT, resulting in the 5-year overall survival (OS) rate of 12%–50% (3, 12). Although surgery is a standard of care for localized MUT, the most appropriate care for metastatic or relapsed cases has not been established. MUT resembles enteric adenocarcinoma histologically and may respond to chemotherapy used to treat colorectal cancer (13). Most of MUT cases expressed CDX2 and CK20 (9, 13, 14), which was also positive in adenocarcinoma of colorectal cancer. Several genomic analyses showed that MUT presented a similar molecular profile with colorectal carcinoma, with a RAS mutation rate of 32%–57% and BRAF mutation rate of 18% (13, 15, 16). But the standard treatment modalities for MUT are lacking. Although the backbone therapy for localized disease remains surgical resection, the systemic therapy for recurrence and metastasis cases is not well known (17). The chemotherapy regimens are also similar to those for colorectal cancer, but the efficacy varies in different reports (4, 18–20). Here, we present the results of a retrospective study of treatment outcome in different chemotherapy regimens in patients with advanced or relapsed MUT in Sun Yat-sen Cancer Center (SYSUCC).

## Materials and Methods

### Patient Selection and Treatment

From January 2014 to September 2020, we enrolled 24 patients with relapsed or advanced MUT at SYSUCC. The study protocol was approved by the ethical committee of Sun Yat-sen University Cancer Center. Eligible patients had histologically confirmed MUT and had adequate organ function apart from organ function affected by disease. Evaluation included. The data reviewed included the patients’ demographics, tumor characteristics, standard laboratory tests, CT scans of the whole body, and the treatment regimens applied. The staging information was based on the 7th UICC TNM Classification (21). Besides, MUT was also staged according to the Sheldon staging system (22), which defines four stages, including I, no invasion beyond urachal mucosa; II, invasion confined to the urachus; III, local extension into bladder (IIIA), abdominal wall (IIIB), peritoneum (IIIC), or viscera other than the bladder (IIID); and IV, metastasis to regional lymph nodes (IVA) or distant sites (IVB).The chemotherapy regimens applied for each patient were decided by experienced oncologists in SYSUCC. The common chemotherapy regimens included gemcitabine (1 g/m2, i.v., d1, d8, q21d), oxaliplatin (130 mg/m^2^, i.v., d1, q21d), capecitabine (1 g/m^2^, po, d1–14, q21d), nanoparticle paclitaxel (260 mg/m^2^, i.v., d1, q21d), and cisplatin (25 mg/m^2^, i.v., d1–3, q21d). All cycles were repeated at 21-day intervals. Treatment was administered until death, progressive disease (PD), unacceptable toxicity, lost to follow-up, or patient or investigator decision.

### Toxicity Evaluation

Adverse events (AEs) were graded according to the Common Terminology Criteria for Adverse Events version 4.0. The relative frequency of each AE considered possibly, probably, or likely related to chemotherapy was estimated as the proportion of all toxicity-evaluable cycles in which toxicity was observed.

### Response Assessment

The objective response was sustained for a minimum of two consecutive imaging evaluations at least 4 weeks apart. Disease was also evaluated using RECIST version 1.1 for response assessment. CT was used to assess treatment response at baseline and after every two cycles of chemotherapy. Follow-up CT scans were performed every 6 months for 2 years or until PD.

### Statistical Analysis

The study population for all analyses included patients enrolled in the study who had an adequate baseline tumor assessment. Descriptive statistics were used to summarize patient characteristics, treatment administration, antitumor activity, and safety. Survival was measured from initiation of therapy until death. The disease control rate (DCR), objective response rate (ORR), progression-free survival (PFS), OS, and AEs were also analyzed. A cutoff date of April 20, 2021, was established for analyzing data for this report. OS and PFS rates were assessed using Kaplan–Meier analyses with SPSS 25.0 software (SPSS Inc., Chicago, IL, USA) and R version 4.0.2.

## Results

Twenty-four eligible patients were enrolled and treated (**Table 1**). Patients were aged from 28 to 69 years, with three patients (12.5%) were aged more than 60 years. Most patients were male (83.3%). All patients received primary surgery. Nineteen patients received urachal excision or transurethral bladder tumor resection, and five patients received partial cystectomy (**Table 1**). Six patients also received pelvic lymph node dissection. Three patients received second surgery after local relapse. No patients received neo-adjuvant chemotherapy. Seven patients received adjuvant chemotherapy after surgery. Fourteen (58.3%) patients were diagnosed at staged III after surgery.

**Table 1:** Characteristics of patients.

Characteristics	*n* (%)
Male sex	20 (83.3%)
Age (years)	
Median (range)	45 (28~69)
TNM stage at diagnosis	
I	1 (4.2%)
II	4 (16.7%)
III	13 (54.2%)
IV	4 (16.7%)
Not applicable	2 (8.3%)
Sheldon tumor stage	
I	1 (4.2%)
II	4 (16.7%)
III	14 (58.3%)
IV	5 (20.8%)
**Initial treatment**	
Surgery with/without radiotherapy or chemotherapy	24 (100%)
Urachal excision or transurethral bladder tumor resection	19 (79.2%)
Partial cystectomy	5 (20.8%)
Radical cystectomy	0
Radiotherapy with/without chemotherapy	0
Chemotherapy	0
**Metastasis site**	
Local relapse	15 (62.5%)
Peritoneal or omental implantation	15 (62.5%)
Lymph node metastasis	11 (45.8%)
Lung	11 (45.8%)
Bone	4 (16.7%)
Liver	3 (12.5%)

The most common metastasis was peritoneal or omental implantation (62.5%) and local relapse of the bladder (62.5%), lung (45.8%), and lymph nodes (45.8%). For first-line systematic chemotherapy, nine patients received XELOX (capecitabine and oxaliplatin), seven patients received TX (paclitaxel and capecitabine), and eight of them received other chemotherapy including GP (gemcitabine and cisplatin), TP (paclitaxel and cisplatin), TN (paclitaxel and nedaplatin), and tislelizumab (**Supplementary Table 1**). Since the regimens were heterogeneous and decided case by case, we compared the survival outcome in the following methods: 1) platinum-based (patients administered cisplatin, oxaliplatin, carboplatin, or nedaplatin) *vs.* non-platinum based; 2) taxol-based (patients received nanoparticle paclitaxel, paclitaxel liposome, or docetaxel) *vs.* non-taxol based; and 3) fluorouracil based (5-fluorouracil or capecitabine) *vs.* non-fluorouracil based. Sixteen patients received platinum-based regimens, 11 patients received taxol-based regimens, and 15 received fluorouracil-based regimens. The remaining one received tislelizumab monotherapy.

**Figure 1:**
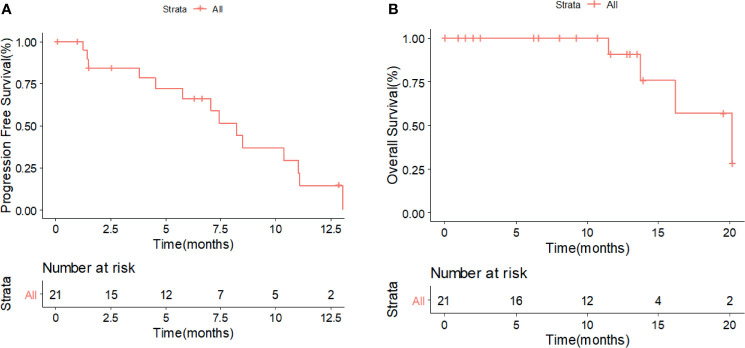
Kaplan–Meier survival analysis for PFS **(A)** and OS **(B)** in all patients with advanced or metastatic MUT. PFS, progression-free survival; OS, overall survival; MUT, malignant urachal tumor.

Overall, only one patient treated with XELOX achieved partial remission (PR), and no patient achieved complete remission (CR); the ORR among all treated patients was 4.2% (1/24). Seventeen patients presented stable disease (SD) after treatment. The DCR for all patients was 75% (18/24). The median PFS and OS were 7.43 and 29.7 months, respectively. The 6-month and 1-year PFS rates were 56.5% and 13.6%, respectively. The 2-year and 3-year OS rates were 57.3% and 19.1%, respectively (**Figures 1A, B**).

**Figure 2:**
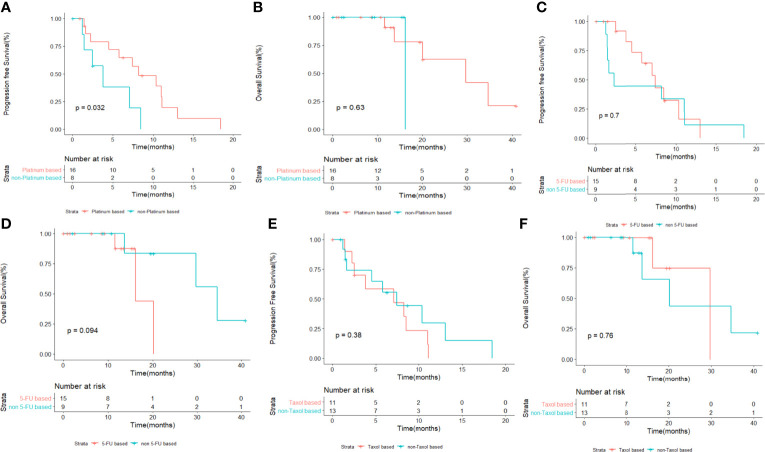
Kaplan–Meier survival analysis for PFS **(A)** and OS **(B)** in patients with or without platinum-based therapy. Kaplan–Meier survival analysis for PFS **(C)** and OS **(D)** in patients with or without platinum-based therapy. Kaplan–Meier survival analysis for PFS **(E)** and OS **(F)** in patients with or without taxol-based therapy. PFS, progression-free survival; OS, overall survival.

**Figure 3:**
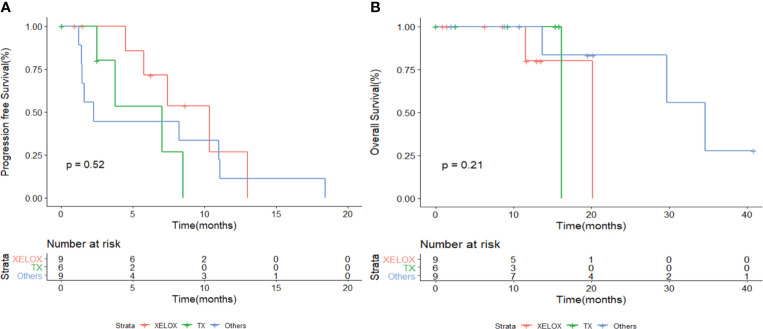
Kaplan–Meier survival analysis for PFS **(A)** and OS **(B)** in patients with different chemotherapy regimens. PFS, progression-free survival; OS, overall survival.

The DCR for patients treated with XELOX and TX as first-line chemotherapy was 100% (9/9) and 83.3% (5/6), respectively. The ORR for patients treated with XELOX was 11.1% (1/9). The median PFS in patients treated with and without platinum-based chemotherapy was 8.23 and 3.80 months (*p* = 0.032), respectively (**Figure 2A**). The 6-month PFS rates in patients with and without platinum-based chemotherapy were 56.5% and 19.0%, respectively. The median OS in in patients treated with and without platinum-based chemotherapy was 29.7 and 16.2 months (*p* = 0.63), respectively (**Figure 2B**). No significant difference was shown for both PFS and OS in patients treated with and without fluorouracil-based chemotherapy (**Figures 2C, D**). The patients treated with non-fluorouracil-based chemotherapy seemed to achieve longer OS (median OS: 34.6 *vs*. 16.2 months, *p* = 0.094). The patients treated with and without taxol-based chemotherapy presented similar median PFS (7.07 *vs*. 7.43 months) and median OS (29.7 *vs*. 20.2 months) (**Figures 2E, F**). The PFS and OS for patients with XELOX, TX, and other regimens revealed no significant difference (**Figures 3A, B**).

**Figure 4:**
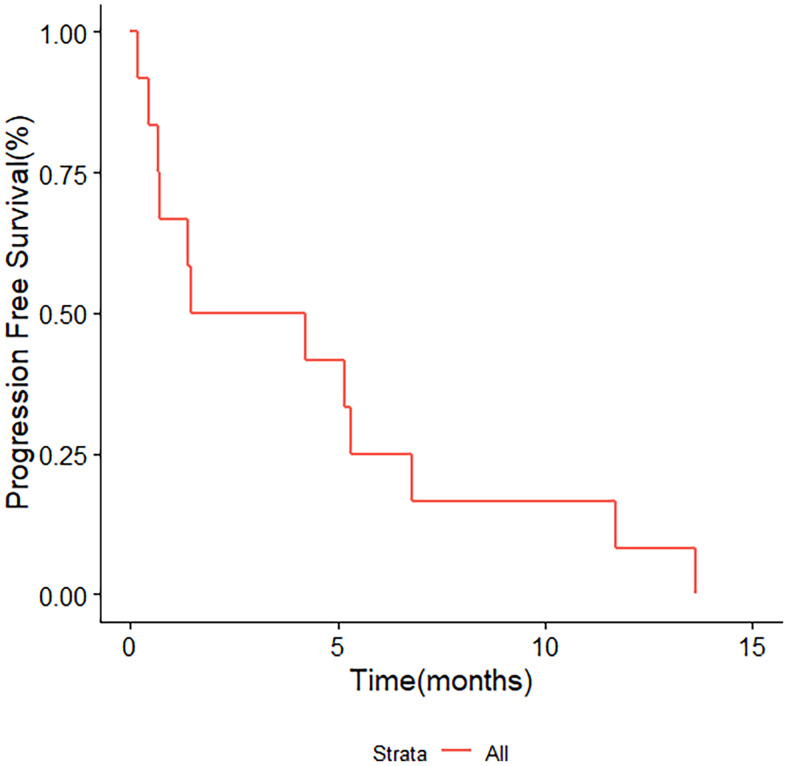
Kaplan–Meier survival analysis for PFS in 12 patients treated with second-line chemotherapy. PFS, progression-free survival.

Among patients who achieved SD or PR, four patients received capecitabine maintenance therapy after combination chemotherapy of XELOX or TX. Two patients remained stable and still received capecitabine till now. Two patients progressed during maintenance at 8.2 and 18.4 months. Twelve patients received second-line chemotherapy after disease progression. The second-line chemotherapy was decided case by case. Two patients received XELOX, two patients received GP, two patients tried a combination of chemotherapy and immunotherapy, two patients received everolimus, and two patients were treated with bevacizumab combined with gemcitabine and nanoparticle paclitaxel. The remaining two patients were treated with irinotecan and capecitabine, and irinotecan and 5-FU (FOLFIRI). A total of five patients received immunotherapy, among which two received tislelizumab, one kind of immune checkpoint inhibitors, as a first-line treatment. A total of three patients received everolimus as second-line or third-line therapy. The median PFS for second-line regimens was 2.85 months (**Figure 4**). One patient achieved PFS for 13.7 months, taking on everolimus monotherapy. The patients were followed up in the outpatient clinic *via* telephone. The median follow-up for all patients was 13.0 months.

The incidences of any AEs and grade III to IV AEs in all patients are summarized in **Table 2**. The AEs for platinum and non-platinum-based regimens are also listed in **Table 2**. The principal AEs were hematological and gastrointestinal events, including leukopenia (70.8%), anemia (70.8%), elevated transaminase levels (33.3%), nausea (25.0%), hand and foot syndrome (16.7%), elevated serum creatinine levels (12.5%), and intestinal obstruction (12.5%). The major grade 3–4 AEs included thrombocytopenia (8.3%) and elevated transaminase levels (4.2%). One patient received changes in treatment of TX instead of TP due to severe intolerant creatinine elevation without progression. No treatment-related death occurred in all groups.

**Table 2:** Summary of adverse events.

	Patients (*n* = 24)		Platinum based (*n* = 16)		Non-platinum based (*n* = 8)	
Events, *n* (%)	Any grade	Grade 3~4	Any grade	Grade 3~4	Any grade	Grade 3~4
Any AE	24 (100%)	3 (12.5%)	16 (100%)	2 (12.5%)	8 (100%)	1 (12.5%)
Hematological toxic effects						
Anemia	17 (70.8%)	0	11 (68.7%)	0	6 (75.0%)	0
Leukopenia	17 (70.8%)	0	7 (62.5%)	0	7 (87.5%)	0
Thrombocytopenia	2 (8.3%)	2 (8.3%)	2 (12.5%)	2 (12.5%)	0	0
Fatigue	2 (8.3%)	0	2 (12.5%)	0	0	0
Diarrhea	1 (4.2%)	0	1 (6.2%)	0	0	0
Dyspepsia	2 (8.3%)	0	2 (12.5%)	0	0	0
Nausea	6 (25.0%)	0	5 (31.2%)	0	1 (12.5%)	0
Elevated transaminases	8 (33.3%)	1 (4.2%)	5 (31.2%)	0	2 (25.0%)	1 (12.5%)
Hand and foot syndrome	4 (16.7%)	0	1 (6.2%)	0	3 (37.5%)	0
Intestinal obstruction	3 (12.5%)	0	1 (6.2%)	0	2 (25.0%)	0
Serum creatinine increased	3 (12.5%)	0	2 (12.5%)	0	1 (12.5%)	0

AE, adverse event.

**Figure 5:**
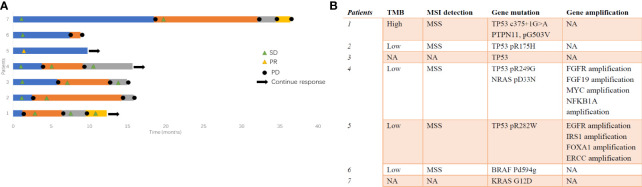
**(A)** The swimmer’s plot for patients with NGS detection and **(B)** summary for the NGS results. NGS, next-genome sequencing.

Seven patients received next-genome sequencing (NGS) test for potential targets (**Figure 5**). TP53 mutation was detected in five patients. One patient reported high tumor mutational burden (TMB), while the others presented low TMB. Patient 1 in **Figure 5** with high TMB presented the best response of SD and PFS of 5.2 months for second-line therapy of TX combined with tislelizumab after progression from tislelizumab monotherapy. Fibroblast growth factor receptor (FGFR) amplification, Myc amplification, ERBB4 amplification, and programmed death ligand-1 (PD-L1) expression of less than 1% was detected in patient 4, with a PFS of 6.53 months for third-line therapy of XELOX and toripalimab after progression from TX and FOLFIRI regimens. Epidermal growth factor receptor (EGFR) amplification was detected in patient 5, with PR after XELOX treatment and undergoing capecitabine maintenance treatment until now.

## Discussion

The carcinoma of the urachus is a rare and aggressive malignant tumor with consequent few data about treatment outcome. We reported the experience in chemotherapy treatment for 24 patients of advanced or metastatic MUT. In our study, patients treated with platinum-based chemotherapy indicated prolonged PFS as compared with non-platinum-based regimens, providing promising options for systemic treatment. Second-line therapy varied in 12 patients, among which everolimus seemed to be effective for the longest PFS. NGS in seven cases revealed a prevalence of TP53 mutation.

Some population-based cohort reported the clinical outcome and prognostic factors in MUT (3, 7, 12). Hager et al. reported 154 and 152 cases of MUT in Germany and SEER database from 2011 to 2015, respectively; the relative 5 year-survival rates were 54.8% in Germany and 64.4% in the United States (7). Another population-based study, which summarized 152 cases of MUT in Netherlands, reported that only 13 out of 45 patients in stage IV received chemotherapy, with poor survival (3). Nagumo et al. reported the clinicopathological features of 456 patients with MUT in Japan (12). In this large retrospective study, it was showed that the most common modality for MUT was surgery alone. However, the chemotherapy regimens for metastatic cases in the article were not available (12). Thus, the proper treatment for metastatic MUT was still unknown. Histologically similar to colorectal adenocarcinoma, a few case reports showed the efficacy for 5-fluorouracil- and cisplatin-based chemotherapy, such as GP and FOLFOX (18, 19, 23). Yanagihara et al. reported modified FOLFOX chemotherapy in five patients with metastatic MUT, resulting in an ORR of 40% and a median OS of 42 months (19). Our study analyzed the first-line chemotherapy of 24 patients, demonstrating that platinum-based regimens were beneficial for patients. The DCR for patients who received platinum-based regimens was 75% (12/16). Most of the patients received oxaliplatin. Both platinum-based and non-platinum-based chemotherapy regimens were well tolerant, with anemia and leukopenia as the most common AEs. In **Figure 3**, it seemed that XELOX presented better PFS but was not statistically significant. Prospective studies are warranted to explore optimal chemotherapy regimens.

Some reports demonstrated that MUT had remarkable molecular similarities to colorectal cancer (24). Colorectal cancers are typified by alterations in several pathways, including adenomatous polyposis coli (APC) loss, the activation of the RAS/MAPK signaling pathway, and TGFβ (by SMAD4 inactivation) pathways (25). Nagy et al. analyzed 40 MUT cases and revealed the prevalence of APC and *PTEN* gene alternation (26). Henning Reis et al. presented 66% of TP53 mutation, 21% of KRAS mutation, 5% of EGFR amplification, and 16% of PD-L1 expression in 70 MUT patients (13). In our study, TP53 mutation was detected in five patients out of seven. We also detected FGFR amplification, EGFR amplification, APC mutation, and KRAS mutation among them. But none of them received anti-EGFR antibody. However, the efficacy of targeted therapy and immune therapy was still not clear. Collazo-Lorduy et al. found that one patient with EGFR amplification and wild-type KRAS achieved 8 months’ response when treated with cetuximab (27). Microsatellite instability (MSI), detected in approximately 15% of all colorectal cancers, is a hypermutable phenotype leading to the loss of DNA MMR activity. MSI-high leads to the accumulation of mutation loads in cancer-related genes and the generation of neoantigens, which stimulate the antitumor immune response of the host, represents a better prognosis and significant association with long-term immunotherapy-related responses (28). In a study of Kardos et al., 25% of urachal tumors harbor inactivating mutations of MMR, MSH6, and MSH2, which might be predictive markers for immune checkpoint blockade (24). One patient with MSH6 mutation resulted in SD after treatment with atezolizumab (24). In our study, most patients were microsatellite stable (MSS). One patient with TMB-high presented more than 5-month PFS when treated with second-line TX and tislelizumab. One patient became SD for 13.7 months when treated with everolimus. Five patients tried different types of PD-1 antibodies, including tislelizumab and toripalimab. However, patients treated with immune checkpoint inhibitors did not present longer PFS and OS than those without immune checkpoint inhibitors. The application of immune checkpoint inhibitors and the biomarkers for prognosis in MUT needs more exploration. It is indicated that a combination of platinum-based chemotherapy with everolimus or anti-EGFR antibody might be promising in the future.

The limitation of this study lies in its retrospective nature and its heterogeneity in baseline risk and treatment factors, which may have led to potential bias. Nonetheless, only seven out of 24 patients underwent NGS, and more genome information is needed in the future. The main strength of the present study was that it analyzed chemotherapy in advanced MUT and showed optimal regimens among the Chinese population. Therefore, prospective clinical trials for this rare disease are warranted for confirmation.

## Data Availability Statement

The raw data supporting the conclusions of this article will be made available by the authors, without undue reservation.

## Ethics Statement

The studies involving human participants were reviewed and approved by Sun Yat-Sen University Cancer Center. The ethics committee waived the requirement of written informed consent for participation.

## Author Contributions

YS and XA designed the study. M-TC, R-QH, M-QN, LL, H-FL, WY, A-QH, and Z-SZ collected the data. CX and M-TC analyzed and interpreted the data. M-TC and CX drafted the manuscript. YS and XA supervised and gave critical revision of the manuscript for important intellectual content. YS and XA provided administrative, technical, and material support. All authors contributed to the article and approved the submitted version.

## Conflict of Interest

The authors declare that the research was conducted in the absence of any commercial or financial relationships that could be construed as a potential conflict of interest.

## Publisher’s Note

All claims expressed in this article are solely those of the authors and do not necessarily represent those of their affiliated organizations, or those of the publisher, the editors and the reviewers. Any product that may be evaluated in this article, or claim that may be made by its manufacturer, is not guaranteed or endorsed by the publisher.

## Abbreviations

PFS, progression-free survival; OS, overall survival; MUT, malignant urachal tumor; SYSUCC, Sun Yat-sen Cancer Center; PD, progressive disease; AE, adverse event; DCR, disease control rate; ORR, objective response rate; PR, partial remission; CR, complete remission; SD, stable disease; NGS, next-genome sequencing; TMB, tumor mutational burden; PD-L1, programmed death ligand-1.
