# Supplementary material for: Treatment Outcome of Different Chemotherapy in Patients With Relapsed or Metastatic Malignant Urachal Tumor
Source: Front Oncol. 2021 Sep 15;11:739134. doi: 10.3389/fonc.2021.739134 (PMC8479186; doi:10.3389/fonc.2021.739134)
Supplement: Supplementary file 1 [file Table_1.docx]

**Supplementary Table 1 Summary of chemotherapy regimens.**

| Regimens |  | *n* (%) |
| --- | --- | --- |
| XELOX | Capecitabine, Oxaliplatin | 8(33.3%) |
| XELOX + trastuzumab | Capecitabine, Oxaliplatin, trastuzumab | 1(4.2%) |
| TP | Paclitaxel, cisplatin | 2(8.3%) |
| TN | Paclitaxel, nidaplatin | 2(8.3%) |
| TX | Capecitabine, paclitaxel | 6(25.0%) |
| TX + tislelizumab | Capecitabine, paclitaxel, tislelizumab | 1(4.2%) |
| Tislelizumab | Tislelizumab | 1(4.2%) |
| GP | Gemcitabine, cisplatin | 3(12.5%) |
